# Editorial: Effects of COVID-19 on sleep and circadian rhythms: Searching for evidence of reciprocal interactions

**DOI:** 10.3389/fnins.2022.952305

**Published:** 2022-08-05

**Authors:** Andrea Romigi, Nicholas-Tiberio Economou, Michelangelo Maestri

**Affiliations:** ^1^IRCCS Neuromed Istituto Neurologico Mediterraneo Sleep Medicine Center, Pozzilli, Italy; ^2^Sleep Study Unit, Department of Psychiatry, University of Athens, Athens, Greece; ^3^Neurology Unit, Department of Clinical and Experimental Medicine, University of Pisa, Pisa, Italy

**Keywords:** sleep, COVID-19, circadian rhythms, sleep-wake cycle, insomnia

The COVID-19 pandemic has had a great impact on people and daily life worldwide since its start in March 2020. To date, numerous studies on different topics have been undertaken to better understand the pathogenicity of this viral infection, develop vaccines and therapy for the COVID-19 disease, and reduce the impact of the pandemic on people, countries, and health. As part of this new and ongoing research, sleep researchers have also shown interest in evaluating the impact of the pandemic on sleep and circadian rhythms, as sleep alterations are often the starting symptoms of health impairment (Merikanto et al., 2021a).

The conditions surrounding “lockdown” and the social-economic consequences connected with them have strongly affected people's sleep, including their sleep-wake cycle and mental health without exception (Brandão et al., 2021; Fränkl et al., 2021; Merikanto et al., 2021b; Partinen et al., 2021). Both adult and adolescent evening-types have developed sleep problems during the pandemic and have had more mental health issues than other chronotypes (Merikanto et al., 2021a; Partinen et al., 2021). Even though increased flexibility in sleep–wake behaviors can lead to benefits, the negative consequences of the pandemic on sleep and mental wellbeing are prominent when compared with supposed positive effects (Brandão et al., 2021). Along with increasing infection numbers, the long-term effects of the disease, known as “long COVID”, have been frequently reported in ~13–80% of subjects (Han et al., 2022). Besides the effects of the pandemic, the role of sleep and circadian rhythms and risk for coronavirus infection, disease severity, and persistent symptoms have been hypothesized and explored. As sleep and circadian rhythmicity have a profound role in physiological functions and mental wellbeing (Bishir et al., 2020), such as the immune system and neural functioning (Richter et al., 2021), disturbances in sleep and circadian rhythms likely play a significant role in the liability of COVID-19 disease and its severity, as well as the risk for developing persisting symptoms. Previous research findings imply that the neurological effects of SARS-CoV-2 infection may also reciprocally increase the risk of sleep and mental health problems, such as nightmares, symptoms of insomnia, and the severity of depression (Boldrini et al., 2021). Several authors have suggested a bidirectional relationship between sleep and COVID-19. In addition, sleep problems are highly evident in different groups (i.e., patients infected with COVID-19, children and adolescents, healthcare workers, and the elderly) (Jahrami et al., 2022).

In their contribution to this collection, Lin et al. explore the presence of nightmares and their association with features of sleep quantity (sleep duration) and sleep quality (sleep efficiency) among health workers, “frontline medical workers” exposed during the COVID-19 pandemic in Wuhan, China. In total, 528 healthcare workers (including 114 doctors and 414 nurses) were enrolled in this study. Sleep characteristics as well as nightmares were assessed by the Pittsburgh Sleep Quality Index (PSQI), while the 12-item General Health Questionnaire (GHQ-12) assessed the general mental health of the subjects. The main finding of the study was that frequent nightmares (taking place at least once/week) were much more prevalent (27.3%) in the studied group compared to the general population. Moreover, frequent nightmares correlated independently with reduced sleep duration (OR = 1.96) and sleep efficiency (OR = 2.17), while subjects with both reduced sleep duration and sleep efficiency were even more associated with frequent nightmares (OR = 2.70). Therefore, reduced sleep duration and/or efficiency in people exposed to traumatic events may underlie nightmares or even PTSD disorders.

In a population of 901 pairs of twins, Tsang et al. investigated the effects of the COVID-19 pandemic and social distancing measures on sleep quality and amount. The authors highlight that sleep reduction (ORs = 2.36 and 3.12 for stress and anxiety, respectively) and poorer sleep quality (ORs = 2.45 and 3.73 for stress and anxiety, respectively) were associated with stress and anxiety, even after taking into account between-family confounds. The timing of the data collection (very early after the WHO declaration of pandemia) and the unique population enable better discrimination of genetic factors. The major strengths of this survey demonstrate that pandemic-related stress and anxiety may be linked to reduced sleep amount and quality.

The paper by Almondes et al. focuses on elderly subjects before and during COVID-19. A group of 914 subjects aged between 65 and 90 years was evaluated using an online questionnaire. The results showed that there was no difference in sleep duration before and during the pandemic, although there was a worsening of some aspects related to sleep such as sleep continuity, with a higher vulnerability in women. Some behaviors may act as protective factors in this population, such as walking and keeping contact with others lessening the effect of isolation. Other social aspects such as financial stability, family support, and a high level of education helped them to cope better with psychological and sleep difficulties in this population. It can be hypothesized that in elderly subjects, the COVID-19 pandemic could impact less than in younger subjects, even if their frailty should not be overlooked.

Another important group of sleep complaints came from patients who suffered from acute COVID-19 infection. Van der Ende et al. compared hospitalized patients with and without COVID-19 to determine the main reasons for sleep disruption. The authors found poor subjective sleep quality and quantity in both groups without significant differences, even if total sleep deprivation was reported five times more often by COVID-19 patients, among whom physical and psychological discomfort and hospital noise seemed to be the main factors. Finally, Giri et al. review recent literature regarding the bidirectional relationship between sleep, circadian rhythms, inflammation, and immunity as crosstalk in health and disease. Throughout this pandemic, low-quality sleep has had a disruptive effect on the immune system at the molecular level, greatly increasing susceptibility to diseases. On the other hand, healthy sleep also makes recovery faster. The authors emphasize rules to improve sleep-wake quality and suggest chronotherapy as an emerging interesting concept, where drugs should be administered to patients in sync with their circadian rhythms, in the management of COVID-19.

## Author contributions

AR, N-TE, and MM contributed to conception and finalization of the editorial. All authors contributed to the article and approved the submitted version.

## Conflict of interest

The authors declare that the research was conducted in the absence of any commercial or financial relationships that could be construed as a potential conflict of interest.

## Publisher's note

All claims expressed in this article are solely those of the authors and do not necessarily represent those of their affiliated organizations, or those of the publisher, the editors and the reviewers. Any product that may be evaluated in this article, or claim that may be made by its manufacturer, is not guaranteed or endorsed by the publisher.
